# A Framework for the Objective Assessment of Registration Accuracy

**DOI:** 10.1155/2014/128324

**Published:** 2014-02-10

**Authors:** Francesca Pizzorni Ferrarese, Flavio Simonetti, Roberto Israel Foroni, Gloria Menegaz

**Affiliations:** ^1^Department of Psychology, Royal Holloway, University of London, Egham TW20 0EX, UK; ^2^Department of Computer Science, University of Verona, 37134 Verona, Italy; ^3^Department of Neurological, Neuropsychological, Morphological and Movement Sciences, University of Verona, 37126 Verona, Italy

## Abstract

Validation and accuracy assessment are the main bottlenecks preventing the adoption of image processing algorithms in the clinical practice. In the classical approach, a posteriori analysis is performed through objective metrics. In this work, a different approach based on Petri nets is proposed. The basic idea consists in predicting the accuracy of a given pipeline based on the identification and characterization of the sources of inaccuracy. The concept is demonstrated on a case study: intrasubject rigid and affine registration of magnetic resonance images. Both synthetic and real data are considered. While synthetic data allow the benchmarking of the performance with respect to the ground truth, real data enable to assess the robustness of the methodology in real contexts as well as to determine the suitability of the use of synthetic data in the training phase. Results revealed a higher correlation and a lower dispersion among the metrics for simulated data, while the opposite trend was observed for pathologic ones. Results show that the proposed model not only provides a good prediction performance but also leads to the optimization of the end-to-end chain in terms of accuracy and robustness, setting the ground for its generalization to different and more complex scenarios.

## 1. Introduction

In medical image registration high quality and accuracy are fundamental due to their impact on the outcome of the clinical task. On top of this, validation is required in order to transfer the scientific research outcomes in clinically usable solutions. However, the discrepancy between intensity of research and lack of robust applications is striking, with the transfer of medical image processing systems from algorithmic development into clinical applications being the major bottleneck.

Validation and evaluation of image registration results are integral components of any image registration method. Despite the importance of validation before clinical use of image registration, regardless in which clinical context it will be utilized, there are today no established criteria or methods for this. This lack of gold standards and adequate validation methods is impeding a wide-spread usage of image registration in different clinical workflows [1]. However, some efforts have been made in this area and there is work going on [2].

In [3], Jannin et al. recommend the development of standardized validation procedures for medical image processing techniques including registration. In particular, validation using a common, publicly available set of validation data with corresponding ground truth is advised. Unfortunately, few such datasets are currently available due to the logistical difficulties of creating a comprehensive reference standard for registration.

The availability of public image databases for experimental purposes allows the validation of computational methods under a common experimental framework. They also permit to reproduce the results claimed by research groups, relative both to diagnostic issues and to computational methods. In this regard, the simulated magnetic resonance (MRI) images from the BrainWeb site [4] and the clinical images from the Internet Brain Segmentation Repository [5], which are provided with expert manual segmentations that can be used as the ground truth for validation, have been widely used as benchmarks for a number of algorithms devoted to segmentation, registration, filtering, and correction of artifacts in MRI. A number of new resources have been added in recent years to those early public database efforts, and new projects have been developed individually by research groups, as the Laboratory of Neuro Image [6]. Resulting from these projects there are many public resources that are available for validation purposes of both clinical conclusions and computational algorithms, keeping pace with the fast evolution of the imaging devices and techniques. Another dataset to be mentioned is the “Vanderbilt Dataset” [7], which is a set of volumetric brain images available online as part of the Retrospective Image Registration Evaluation Project. The reference standard for registration of these images is based on skull-implanted markers. A further set of 16 brain MR images is available from the Non-Rigid Image Registration Evaluation Project [8] along with segmentation information.

The literature on registration evaluation methods may, in general, be divided into no-reference methods which do not rely on a reference standard [9] and full-reference methods which do. However, the standards are often not accessible to most researchers [10].

Glatard et al. [11] proposed a technique for estimating the ground truth with a “bronze standard” obtained through the application of many registration algorithms to a large database of images. However, the method has not been extended to nonrigid registration and is very dependent on having a large number of good registration algorithms as well as an extensive database, both of which are difficult for many researchers to obtain.

Another classical method used for registration evaluation consists in measuring the overlap of structures of interest in the target and registered images [12], though, this requires the availability of segmentations of the structure(s) to be considered. Although overlap-based evaluation is intuitively reasonable, it should be observed that its performance is bounded by the quality of the reference segmentations which in turn depends on the type of structures that are considered.

Other authors proposed to measure registration accuracy based either on point sets [13] or on manually annotated contours or landmarks [14]. Manual annotations are frequently small sparse point sets with poor distribution throughout the image, which leads to poor results on deformable registrations, while they represent a viable solution for rigid and affine transformations. Larger sets of landmarks have been used by some authors, most notably Castillo et al. [10]. However, these are typically required to be carried out by an expert observer which is expensive and impractical for a large set of images. The problem becomes increasingly difficult when addressing the case of abnormalities and/or highly compressible and elastic tissues, having inherent nonrigid behaviour and for which solid anatomical landmarks are missing [15].

In this paper the approach proposed in [13] is followed to investigate registration performance by synthetically aligning data such that the original image and the transformed image are known in advance as well as the ideal transform between them. With this approach there is no need for a reference standard because it is already implied by the synthetic transformation used.

In particular, an approach following a novel perspective relying on Petri nets (PN) [16] is proposed here. Instead of evaluating *a posteriori* the effectiveness of a given registration algorithm, the critical steps that are involved in the complete imaging chain are identified *a priori*. This has the unique advantage of providing a prediction of the performance with respect to the feature of interest (accuracy) as well as a framework for the run-time dynamic monitoring and optimization of the considered processing pipeline.

In the specific scenario considered here, validation and accuracy prediction for MRI rigid and affine registration algorithms are considered. With the increasing availability of MR scanners, more and more patients have repeated examinations, and radiologists are frequently asked to interpret multimodal information and report on changes that reflect the subjects progression or regression and may require a change in ongoing treatment or the start of a new one. We have employed rigid and affine registration techniques to combine information from multiple modalities and monitor changes in the brain in individual subjects who underwent serial MRI examinations. This approach other than allowing accurate diagnosis and highlighting disease progression and response to treatment to be monitored with great sensitivity fits naturally with the noninvasive nature of MRI. A feature of serial studies on individual subjects is that the images obtained at each examination are likely to show the same anatomic regions only relatively misaligned. This makes the problem of determining the transformation that maps one image to another much easier to solve. However, similarity of images and the requirement to detect changes with maximum sensitivity mean that the spatial match can and must be to subvoxel precision, and the voxel intensity values in the final registered images must have minimal interpolation errors [17].

We consider such a context well suitable for highlighting the potential of the proposed objective assessment framework. Since the number of control parameters is kept small, the role of each parameter can be disambiguated and checked. Furthermore, the flexibility and openness of the proposed framework allows the inclusion of other control parameters eventually intervening in the scenario under investigation as well as its transposition to other pipelines.

The methodology is validated on both synthetic and real MRI data of the human brain including both healthy and pathological cases. Furthermore, the suitability of synthetic data for training the PN for further use on clinical cases is investigated. This allows shaping the PN on a controllable dataset avoiding the inherent variability of real data.

This paper is organized as follows.  Section 2 illustrates the PN-based paradigm; Section 3 provides an overview on the clinical problem and presents the registration pipeline and the datasets. In Section 4, the performance of the proposed approach is assessed and in Section 5 discussed, while providing some examples of other applications and clinical scenarios.  Section 6 derives conclusions.

## 2. The Petri Net Paradigm

The connection between PN-based formal methods and image processing validation procedures comes from the business [18] and technical [19] strategy of risk analysis. Preliminary risk analysis or hazard analysis is in fact a qualitative technique which involves a disciplined analysis of the event sequences which could transform a potential hazard into an accident. In this technique, the possible undesirable events are first identified and then analyzed separately. For each undesirable event or hazard, possible improvements or preventive measures are then formulated. In fact the main evaluation steps in a risk analysis procedure are (i) identifying threats and (ii) estimating and (iii) managing risk.

The result from this methodology provides a basis for determining which categories of hazard should be looked into more closely and which analysis methods are the most suitable. Such an analysis also proves to be valuable in working environments where activities lacking safety measures can be readily identified. With the aid of a frequency/consequence diagram [20], the identified hazards can then be ranked according to risk, allowing measures to be prioritized to prevent accidents.

In this work, we propose an alternative interpretation of the PNs such that the framework developed adapts to the problem at hand. In particular, the so-called *hazards* map to the *inaccuracies* that each processing step generates, while the *accidents* are identified as *erroneous* or *not reliable results*, because of showing too low accuracy to be used in the clinical practice. We will rank our processing steps according to their impact in terms of inaccuracy.

**Figure 1:**
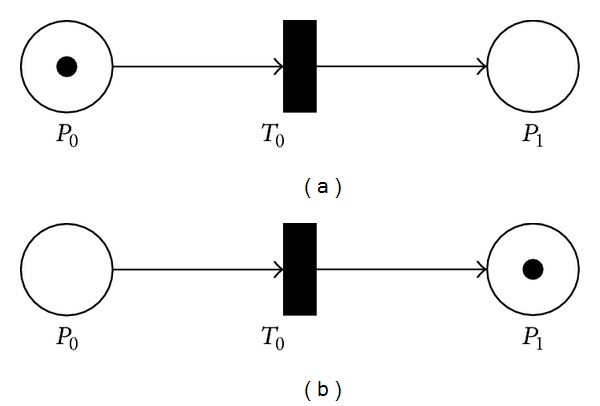
The classical PN consists of places (*P*_0_-*P*_1_), transitions (*T*_0_), and tokens (black dots). The figures visualize two different markings of the same PN, that is, two *different distributions of the tokens*.

More formally, a PN is a tuple (*P*, *T*, *F*) where *P* is a finite set of places, *T* is a finite set of transitions (*P*∩*T* = *⌀*), and *F*⊆(*P* × *T*)∪(*T* × *P*) is a set of arcs (flow relation). From the graphical point of view, a classical PN is composed of three primitive concepts: places, transitions, and tokens (see Figure 1).

Transitions are active components. They model activities that can occur (the transition fires), thus changing the state of the system (the marking of the PN). Transitions are only allowed to fire if they are enabled, which means that all the preconditions for the activity must be fulfilled (there are enough tokens available in the input places). When the transition fires, it removes tokens from its input places and adds some at its output places. The number of tokens removed/added depends on the cardinality of each arc. The interactive firing of transitions in subsequent markings is called token game. The aforementioned formalism is just a classical PN [21]. There are many extensions of PN, each having its own properties in order to model specific real situations, like coloured PN [22] or timed PN [23].

As mentioned above, PNs have classically been used to model concurrency in distributed systems. Here, a reinterpretation of this paradigm is proposed. *Processing units* within the imaging chain correspond to *places; image features* (i.e., acquisition type) and *processing methods* (i.e., metrics, interpolations) correspond to *transitions*. Arcs connect places and transitions. Places may contain a number of tokens that can be chosen according to the net requirements. Tokens are associated to the inaccuracy introduced by each processing method, or step. As a consequence, every path in the net represents a possible combination of parameters that are responsible for the inaccuracy of the results. Bearing these considerations in mind the classical concept of PN was reinterpreted by adding a special state that is used as an *inaccuracy counter*, gathering the tokens from the transitions, that is, critical parameters of the registration, while the token game proceeds along the whole net. In this way the PN has been converted to a weighted graph for which information about the global cost of each path can be calculated. The PN was then calibrated by determining the weights to be assigned to each transition. The solution to this issue falls within the field of the inverse shortest path problems [24], which consist in inferring the weights of the arcs from the global costs of the path by solving the corresponding least square problem. In this process, the metrics mentioned above allow to measure the contributions to global inaccuracy due to each transition (i.e., noise, imaging modality, metric, interpolation, type of transformation, etc.). The minimization of the difference between the predicted and measured global error following the linear least square method through the Moore-Penrose pseudoinverse was exploited to this end.

The choice of using PNs naturally rises the issue of the advantages brought by this solution with respect to neural networks. The reason of the choice of PNs instead of neural networks is twofold. First, PNs enable the run-time monitoring of the pipeline, such that the process can be stopped as soon as the global accuracy becomes unacceptable for the task at hand. Second, neural networks are “black boxes” in the sense that the nature and impact of the factors that rule the mapping of the input to the output are hidden in the training phase and, in general, it is not accessible to the user. Furthermore, the PN-based solution provides and open and flexible framework that can easily integrate new control parameters and be declined for use in different scenarios.

In addition, neural networks are efficient only if the predicting variables are chosen with care, and they are not able to handle categorical variables, presenting many values that can be assigned. Moreover they need a training phase, whose aim is to fix the weights of each single neuron and this phase could be really time consuming, especially if the number of records and variables is huge. Finally, there are not any theorems or models that could define the optimal net; therefore the outcome of a net relies mainly on the experience of its designer.

## 3. Materials and Methods

### 3.1. Data

**Figure 2:**
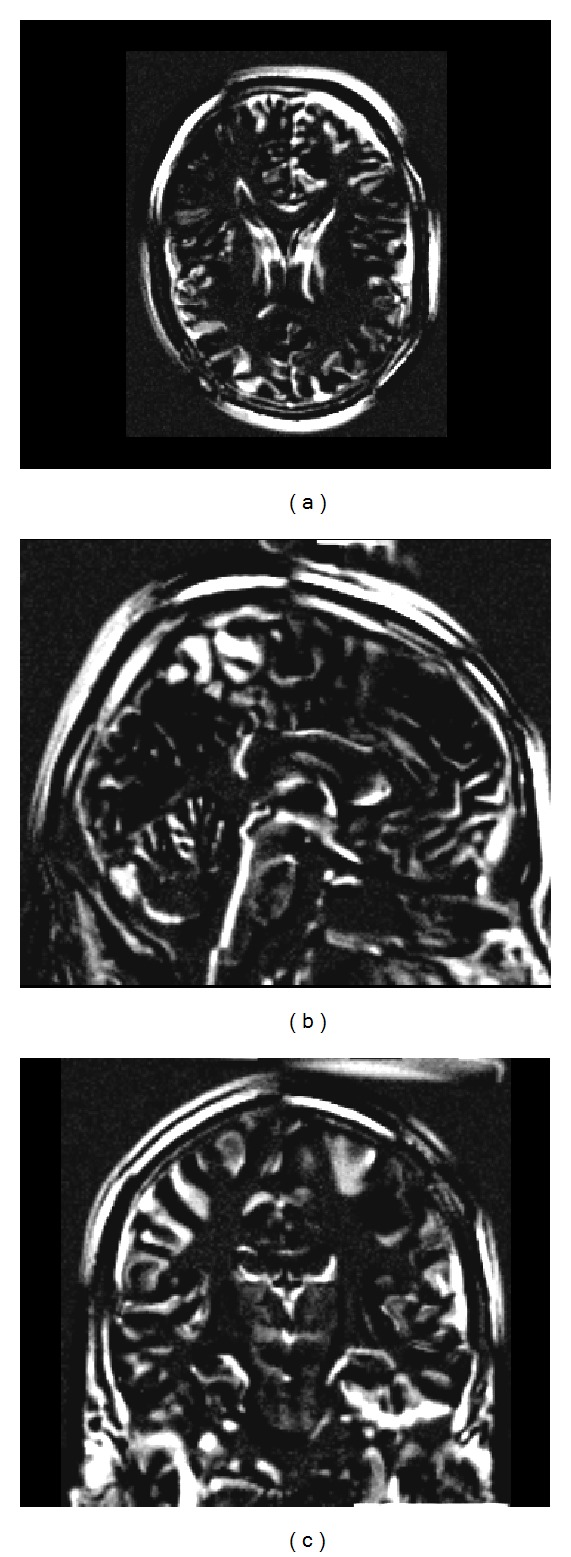
Image difference between the original T1-weighted BrainWeb volume and one of the supplemental volumes generated through a thin plate spline-based three-dimensional deformation grid. (a) Axial (b) sagittal, and (c) coronal views.

The behaviour of the different registration pipelines was assessed on both simulated and real data. Simulated T1- and T2-weighted [25] MRI data volumes of normal brains were obtained from the BrainWeb dataset [4]. These consist of 181 × 217 × 181 voxels of 1 mm^3^ featuring 20% of intensity nonuniformity. These images were registered to the same origin. In order to increase the number of simulated datasets to be used for testing while preserving the imaging features, a thin plate spline-based three-dimensional deformation grid with random displacement was applied to each dataset. The maximum displacement was set to 2 mm in each direction to obtain a sample of brains with global characteristics falling into the range of variation of normal adult brains [26]. It is worth mentioning that the transformation was not constrained to be representative of realistic intersubject local anatomical variations because the goal was to check the robustness of the proposed method with respect to random variations. On top of this, models of intersubject anatomical changes are not available. However, the suitability of the proposed framework to real clinical scenarios is recovered by the characterization of its performance on real data. In this way, 10 additional T1- and T2-weighted datasets were generated.  Figure 2 shows one example of image difference between the original BrainWeb T1-weighted dataset and one of the supplemental volumes generated as described.

**Figure 3:**
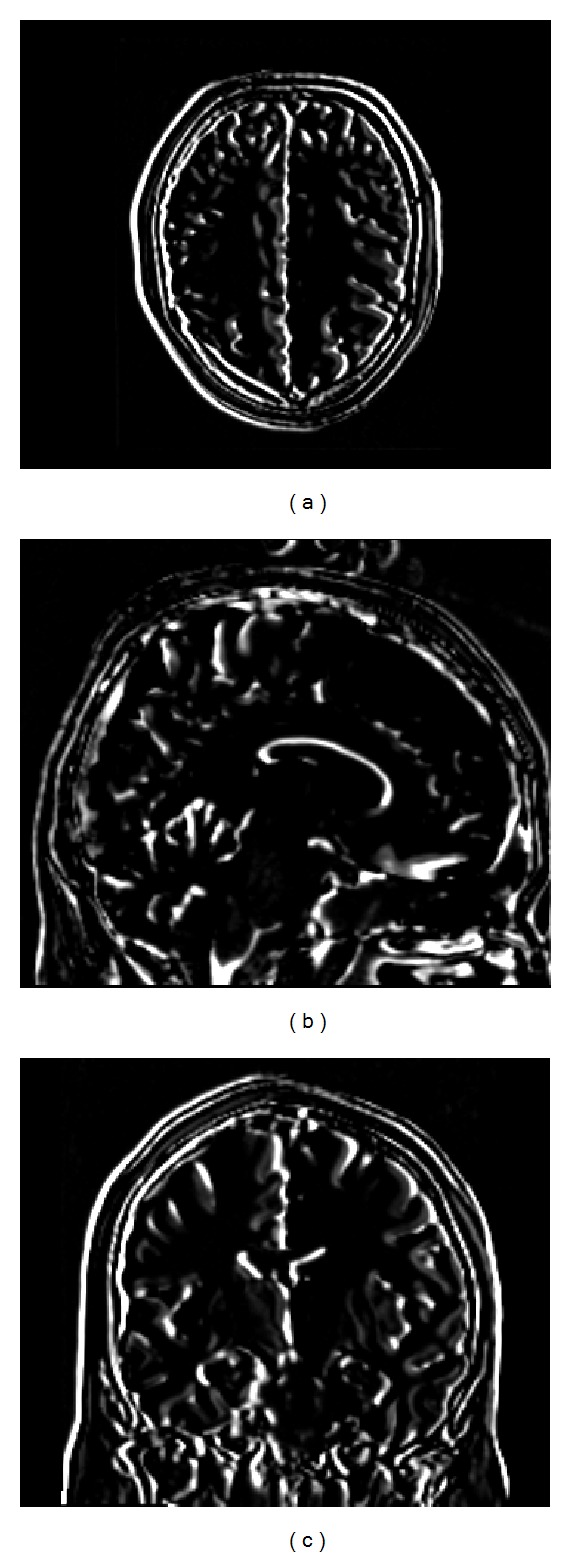
Image difference between one of the T2-weighted simulated volumes before and after the simulated misalignment. (a) Axial (b) sagittal, and (c) coronal views.

T2-weighted volumes were then resampled in order to simulate a plausible misalignment between different acquisitions [27].  Figure 3 shows the image difference between one of the T2-weighted simulated volumes before and after the simulated misalignment.

As for the real data, the images provided by the National Alliance for Medical Image Computing [28], freely downloadable at MIDAS website [29], were used. These consist of 2 datasets.

The first dataset comprises 20 cases out of which 10 are from normal controls and 10 were acquired from patients affected by schizophrenia. Structural MRI was acquired using a 3T (E, at BWH in Boston, MA, USA) equipment. An 8-channel coil was used in order to perform parallel imaging using Array Spatial Sensitivity Encoding techniques with a SENSE-factor (speed-up) of 2. The structural MRI acquisition protocol included two MRI pulse sequences. The first resulted in contiguous spoiled gradient-recalled acquisition (fastSPGR) with the following parameters: TR = 7.4 ms, TE = 3 ms, TI = 600, 10 degree flip angle, 25.6 cm^2^ field of view (FOV), and matrix = 256 × 256. The voxel dimensions were 1 × 1 × 1 mm. The second-XETA (eXtended Echo Train Acquisition) produced a series of contiguous T2-weighted images (TR = 2500 ms, TE = 80 ms, 25.6 cm^2^ FOV, and 1 mm slice thickness). Voxel size was 1 × 1 × 1 mm as it was the case for the simulated data.

The second dataset includes images for 2 autistic children and 2 normal controls (male and female) scanned at 2 years with followup at 4 years from a 1.5 T Siemens scanner. All scans were conducted on a 1.5 T Siemens scanner. Sequences include: a T1-weighted inversion recovery magnetization preparation sequence with a slice thickness of 1.5 mm in the coronal plane, TE = 5.4 ms, TR = 12.3 ms, FOV = 20 cm, flip angle = 40° and 256 × 192 matrix. T2-weighted fast spin-echo images were acquired with the following parameters: 3.0 mm coronal slices, TE = 17/75 ms, TR = 7200 ms, FOV = 20, 256 × 192 matrix.

The alignment between T1- and T2-weighted volumes was evaluated with the help of an experienced radiologist, in order to decide whether the original images were already aligned enough to act as reference images or if it was necessary to proceed with a registration supervised by the radiologist. This validation led us to use the provided T2-weighted as the ground truth for our analysis. As for the synthetic data, the misalignment that can occur while acquiring the data was simulated by applying a random but constrained transformation to the T2-weighted images.

### 3.2. Registration Paradigm

Given a fixed and a moving image, the registration problem is the process of finding an optimal transformation that brings the moving image into spatial alignment with the fixed image. The main difficulty is that the problem is ill-posed, which means, for example, that it might not have a unique solution. The input data to the registration process are two images: the fixed *f*(*X*) and the moving image *m*(*X*), where *X* represents a position in *nD* space. The mathematical formulation of the registration problem involves finding the optimal geometric transformation which maximizes the correspondences across the images. The transform operator *T*(*X*) represents the spatial mapping of one image to the other and the interpolation allows dealing with nongrid positions in the matching process. A metric *S*(*f*, *m*∘*T*) provides a measure of how well the fixed image is matched to the transformed moving image. This measure represents the quantitative criterion that has to be optimized over the search space defined by the parameters of the transformation.

The chain allows many degrees of freedom concerning the choice of the blocks. Each one will have an impact on the overall accuracy. Accordingly, a given set of plausible configurations was analyzed focusing on the considered case study. This set is not intended to be exhaustive, that is, accounting for all the possible parameters that need to be tuned in a real scenario. In fact in this context we are considering only those elements that are actually exploited when multimodal intrasubject registration is performed.

The contribution in terms of inaccuracy of each element was evaluated and used for training the PN, as it will be described in Section 2. In brief, the building blocks of the registration chain are the metric for the distance function *D*, the transformation *T*, the optimization method Opt, and the interpolation filter IF.  Table 1 summarizes the different solutions that were considered.

As it is highlighted in Table 1, the only distance function that is considered here within the registration process is the MI. The reason of this choice is that, to the best of our knowledge, MI is the most suitable to multimodal intrasubject registration. Other standard distances such as cross correlation and mean square error, that cannot account for differences in intensities due to different acquisition modalities, result in worse performance. The performance of the registration pipeline in the different set-ups was evaluated according to four different criteria: (i) normalized mutual information (NMI) (ii) normalized root-mean-square of intensity differences (NRMS) (iii) edge overlap (EO), and (iv) maximum geometric error (MGE). Image similarity and overlaps are widely used as surrogates for image registration accuracy. However, their use is only appropriate for intrasubjects affine registrations, where the misalignment that has to be accounted for is limited and there are no relevant changes in the anatomy. In fact, when used for the evaluation of nonrigid registration, the information they provide is not reliable [30].

Besides the classical metrics, the MGE, that we also call *landmark-based* metric, was introduced to provide the clinician with an index that could be directly mapped to the image space. To this end, 16 markers were randomly placed on each volume to be registered along the axial, sagittal, and coronal directions, respectively. We have taken the MGE as the metric of our choice, since it represents the worst fitting landmark distance, which is a conservative estimate of the real misalignment.

Since the datasets were perfectly aligned, an arbitrary misalignment was introduced by means of an orthogonal transformation matrix corresponding to a rigid transformation [27].

The applied transformation intended to model a simple displacement mimicking the result of the head movements that can occur during an MRI acquisition. Accordingly, a linear transformation **d**(*x*, *y*, *z*) : *R* ^3^ → *R* ^3^ simulating translations and rotations of the head was applied. The **d** transformation matrix was defined using two parameters *t*_trans⁡_ and *t*_*rot*⁡_ ruling the translations and rotations along the three axes *x*, *y*, and *z*, respectively. The range of variation for *t*_trans⁡_ was from a minimum of 0 mm to a maximum of 25 mm in each direction, while *t*_*rot*⁡_ ranged between −5° and +5° around each axis. The values were selected randomly for each dataset.

Then, markers were placed on the original images and projected onto the transformed ones. Let {*PS*_*i*_^*k*^}, *i* = 1,…, *m* be the markers placed on the original image *k*, and let {*PS* *t*_*j*_^*k*^} be the set of markers located on the image generated by applying the transformation matrix to image *k*. The transformation **d**(*x*, *y*, *z*) that was applied to the images as described above was also applied to the set *PS*_*i*_^*k*^ resulting in the additional set {*PS* *t*_*j*_^*k*^}. Accordingly, two corresponding point sets were defined for each image: {*PS*_*i*_^*k*^} and {*PS* *t*_*j*_^*k*^}, respectively.

Finally, the previously calculated registration matrix *T* was applied to {*PS* *t*_*j*_^*k*^} leading to a third points set *T*({*PS* *t*_*j*_^*k*^}). By measuring the Euclidean distance between {*PS*_*i*_^*k*^} and *T*({*PS* *t*_*j*_^*k*^}) for all the corresponding points **x**_*i*_ and **y**_*i*_, the MGE error measure was obtained as the maximum distance between each pair of corresponding points in the two sets as follows:

(1) $$\begin{array}{c} \text{MGE}\left(\left\{PS_{i}^{k}\right\},T\left(\left\{PSt_{j}^{k}\right\}\right)\right)=\max\left\{D_{E}\left(x_{i},y_{i}\right)\right\}\\ x_{i}\in \left\{PS_{i}^{k}\right\},\;y_{i}\in T\left(\left\{PSt_{j}^{k}\right\}\right),\;i\in \left\{1,2,\ldots ,n\right\}, \end{array}$$

where *D*_*E*_ represents the Euclidean distance.

### 3.3. Noise Modeling

For a fair comparison of the results obtained on simulated and real data, respectively, noise was added to the simulated ones. To this end, the level of noise affecting real data was first estimated. The image intensity in MR magnitude images in the presence of noise is shown to be governed by a Rician distribution [31]:

(2) $$\begin{array}{c} p\left(x\;|\;\nu ,\sigma_{n}\right)=\frac{x}{\sigma_{n}^{2}}\exp\left(-\frac{x^{2}+\nu^{2}}{2\sigma_{n}^{2}}\right)I_{0}\left(\frac{\nu x}{\sigma_{n}^{2}}\right), \end{array}$$

where *σ*_*n*_ is the standard deviation of Gaussian noise in the complex domain, *ν* is the amplitude of the signal without noise, *x* is the value in the magnitude image, and *I*_0_ is the zero order modified Bessel function [32]. Based on this, noise was estimated as follows. First, 4 random regions were extracted from the background of T2-weighted real datasets. Second, nonlinear minimization was applied to the intensity histograms of the regions to perform multivariate fitting to the model. For the cost function a moment-matching method was used. In this way, the noise parameters *σ* and *ν* were estimated.

## 4. Results

**Figure 4:**
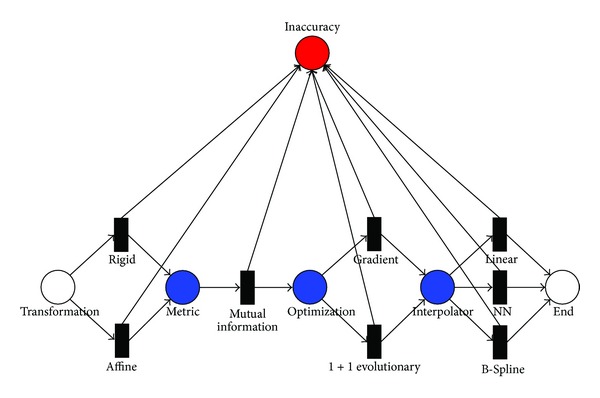
PN focusing on the critical aspects that can affect the overall accuracy of the registration process.

**Figure 5:**
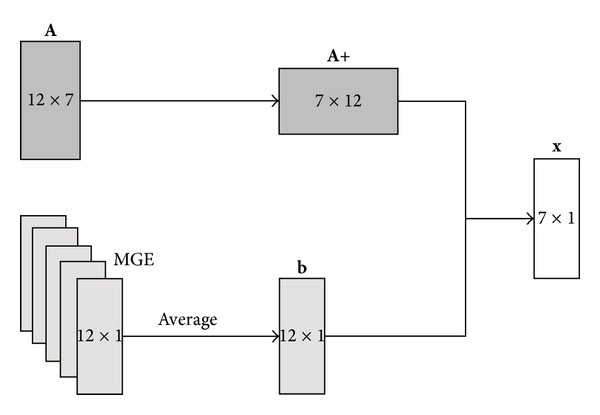
two-steps process for obtaining matrix **x** from the pseudoinversion of **A** and the measured MGEs.

For easiness of notations, simulated and real data are labelled as SIM and *R*, respectively. Both sets were split in two subsets of half the numerosity each, to be used for training and testing. For training data, the superscript *τ* was used, while *t* identified the testing subset. Furthermore, real data from control patients were assigned the subscript *c* to distinguish them from the pathological ones, identified by *p*.  Table 2 summarizes the labels that were assigned to the different data types. All the critical aspects in the processing workflow that would potentially lead to inaccuracies were considered and the overall process was formalized following the PN paradigm (Figure 4). The inaccuracy level was encoded in the number of tokens collected in a specific state/counter that the registration process reached at the end of the complete workflow (red-state). As mentioned above, the critical points that could affect the final results were assigned to each processing step (blue-states). For each training dataset *τ*, 12 different registrations were carried out and the corresponding MGE values were calculated as in (1). In order to calibrate the transitions of the PN, that is, to assign a weight to each arc that goes from a specific transition to the inaccuracy counter, a linear system must be solved, as illustrated in Figure 5. The goal is in fact to estimate **x**, which encodes the contribution of each control variable to the derived inaccuracy (MGE). In the figure,

- **A**  is the binary 12 × 7 system matrix (where 7 is the number of the registration parameters/components and 12 represents the possible paths along the PN. We are not accounting for the metric selection step here, since the only choice available is represented by the MI, whose impact is therefore not separable);
- **x** is the 7 × 1 vector of the weights;
- **b** is a unique representative 12-elements error vector obtained by averaging the MGEs measures for *τ* datasets.

Since in this case **A** is not full rank, it cannot be directly inverted and the system is ill-posed. In consequence, the Moore-Penrose pseudoinverse was calculated. This method leads as close to the correct inverse as possible in the sense that, out of all the matrices *Z* that minimize

(3) $$\begin{array}{c} {||AZ-I||}_{F}, \end{array}$$

*Z* = *A* ^†^ also minimizes the Frobenius norm ||*Z*||_*F*_. It turns out that these minimization properties also define a unique pseudoinverse even if *A* is rank deficient, which is our case. The weights vector, **x**, was then obtained by composing the pseudoinverse of **A** with **b**. The process for calculating **b** from the measured MGEs is summarized in Figure 5.

Table 3 provides the comparison between the weights obtained from datasets *τ* and *t*, for simulated and real datasets, respectively. The negative weights are not to be considered as “real” values but as the results of the inverse shortest path solution. By definition, the single values represent the contribution of single control variables to the derived inaccuracy, while their sum along a path is the MGE calculated for the chosen registration workflow and is measured in mm.

Accordingly, Table 3 reveals that there is no clear and significant difference between the results obtained by applying a rigid or an affine transformation. This suggests the adoption of the rigid registration, since the impact of the two transformations on the MGE is quite similar (as revealed by the fact that the respective weights are very close one to the other), while being computationally more effective.

Considering the optimization step, all the datasets are coherent in saying that this is a critical aspect in the registration pipeline. The results highlight the wide gap between the gradient descent approach, which is deterministic, and the evolutionary strategy, which is stochastic. This could be due to the fact that the gradient descent reaches at each step a suboptimal solution following the line search direction, while the evolutionary method is not necessarily optimized at each step and therefore it does not have a leading search direction.

Regarding the choice of the interpolation method, while the difference in terms of error between the linear and the B-Spline is not significant, NN interpolation appears to negatively impact on the accuracy of the registration. In fact, the NN interpolation is usually expected to produce the worst results, especially in terms of registration accuracy and grey-level discontinuities due to the simplistic interpolation scheme. Moreover, NN is not recommended in our scenario because it does not provide subvoxel registration accuracy. Overall, this led to the conclusion that the weights are quite independent of the data used on training, opening the way to the use of simulated data to this end.

**Figure 6:**
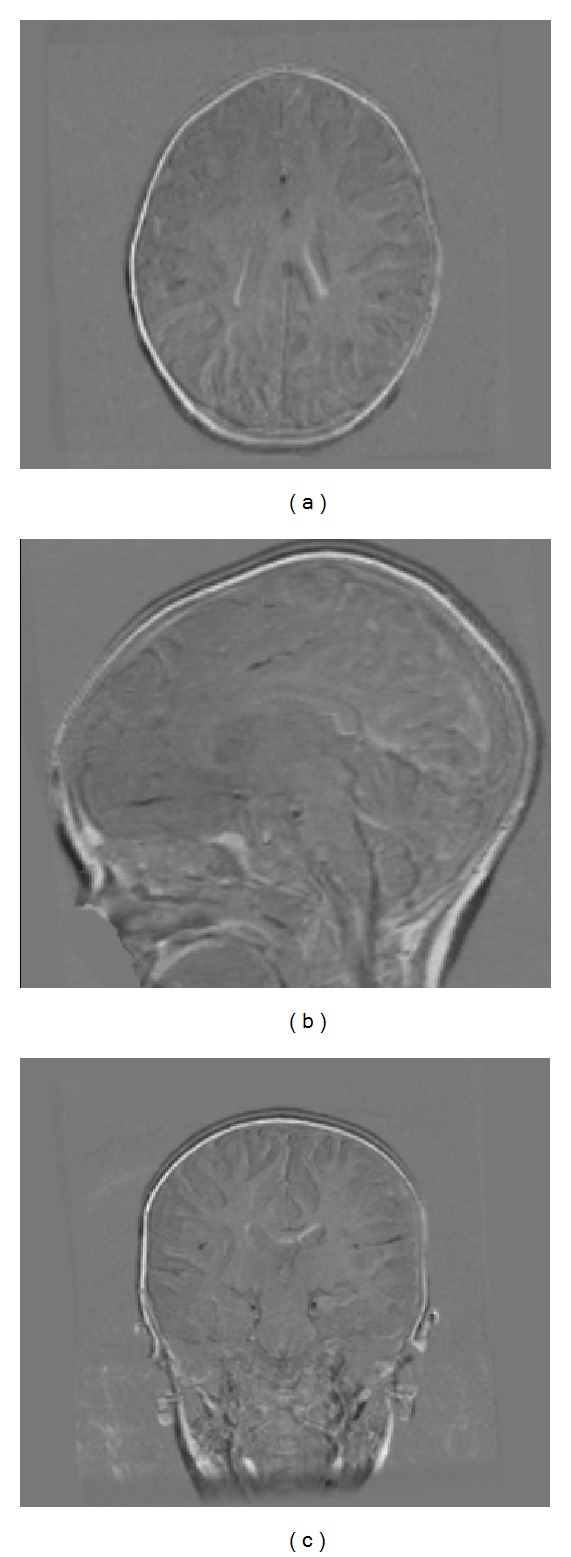
Image difference between the T1-weighted volumes for one of the subjects scanned at 2 and 4 years old. (a) Axial (b) sagittal, and (c) coronal views.

As a proof of concept, the performance of our framework has been evaluated in a more complex scenario, namely, the longitudinal image registration. To this end the same registration methods were applied to T1- and T2-weighted images of 2 autistic children and 2 normal controls scanned at 2 years with followup at 4 years. Postnatal brain development is notably protracted and involves considerable changes in cerebral cortical, subcortical, and cerebellar structures, as well as significant architectural changes in white matter fiber tracts.  Figure 6 shows the image difference between the MRI acquired on one subject at 2 and 4 years old.

While the weights for the interpolation and optimisation parameters show similar impacts to the overall outcome as it was the case for adult real and simulated data, the choice of the registration type here has a much stronger effect. The inaccuracy of a rigid registration is in fact 23% higher than that due to the affine registration (with a 1% difference between autistic and control subjects). This supplementary evaluation shows the flexibility of the framework developed, paving the way to the exploitability of our validation approach to diverse and more complex clinical scenarios.

Robustness was assessed by calculating the Pearson's correlation coefficient *ρ* between the PN weights resulting from the use of different sets of volumes as training and testing sets, respectively.

The Pearson's correlation for the 10 simulated datasets was on average 0.995, while for the real datasets it was 0.999 and 0.977 for healthy and pathologic subjects, respectively.

To obtain a quantitative assessment of such observed lack of bias due to the training data, each dataset was split in a training (*τ*) and testing (*t*) sample, as introduced earlier in this paper. These were used for (i) calculating the weights and (ii) evaluating the prediction error, respectively.

**Figure 7:**
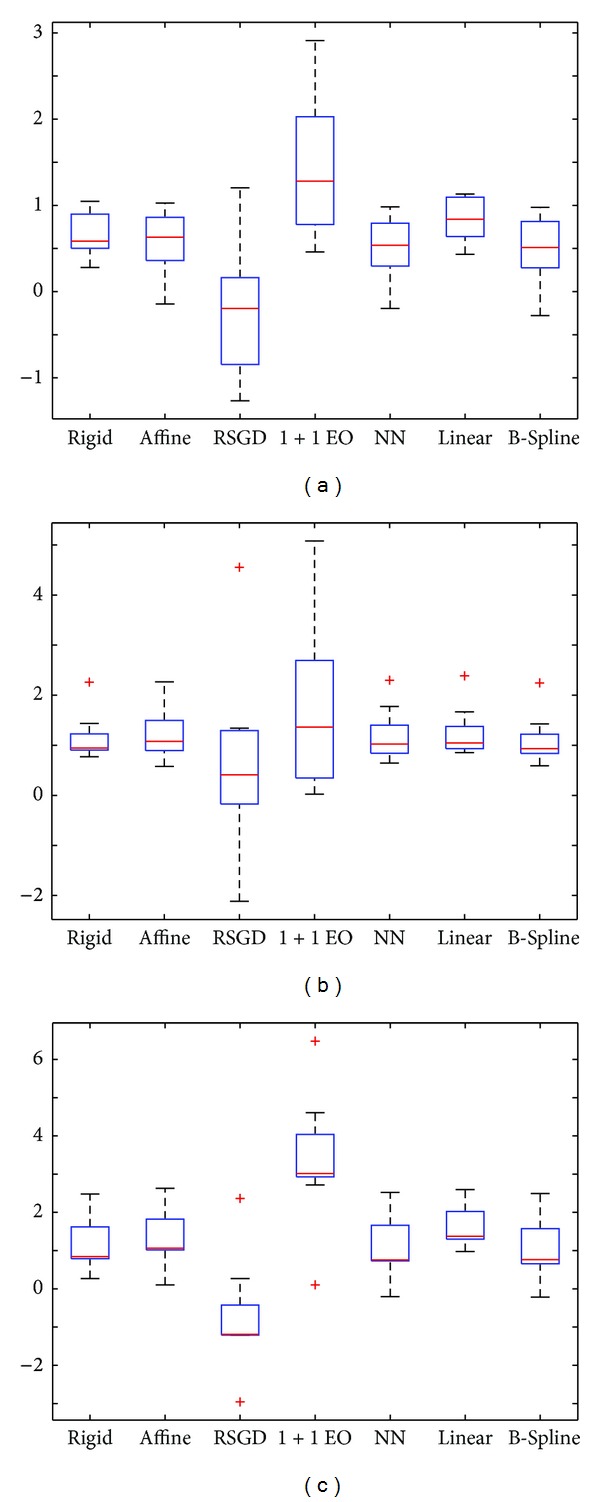
Box plots of the weights for the single processing steps. (a) Real control subjects *R*_*c*_; (b) real pathologic subjects *R*_*p*_; (c) simSSSSulated data SIM.

To evaluate the predicting power of the PN when trained on simulated data, the correlation between the estimated and the measured values of accuracy was assessed on real images. In Table 4 the correlation between the weights obtained using different *τ* and *t* datasets is shown, highlighting the fact that the simulated data have a high predicting value for the real data in terms of absolute registration error. The box plots in Figure 7 regarding *R*_*c*_, *R*_*p*_, and SIM datasets illustrate both central tendency and dispersion of the resulting set of weights. These were calculated on training and testing data together. The trends for both the simulated and the real data are similar, therefore demonstrating the robustness of the registration method with respect to the dataset type (simulated versus real and healthy versus pathologic). These results justify the exploitation of simulated data to gain first insights on the behaviour of a registration system on real data.

However, the pattern as well as the dispersion in correspondence of the optimization method for the simulated data are different from those observed on real data. This is probably due to the noise. In fact, the noise estimation assumes that the original background is homogeneous and signal free. This assumption makes the estimate sensitive to errors and bias artifacts. This plot also reveals that there is a close similarity between the measures obtained from healthy and schizophrenic subjects, respectively. This suggests that this pathology does not show evident anatomical abnormalities that therefore could impact the registration process. Concerning the outliers, they only appear on the optimization step for the simulated data, while there is a pathologic subject that shows net weights not consistent with the other datasets. Investigating more in details the raw data, the presence of slight acquisition artifacts was detected on the images that could have affected the subsequent registration process. In Figure 7(c) it is also possible to notice that the distributions for all the weights are negatively skewed, while for the real data they are more symmetric. This behaviour could be due to the noise added on the data or to the way the data themselves were generated. It would be of interest for future works to investigate the impact of different noise modeling techniques on different simulated datasets on weights' distribution shapes.

Then, the correlation, or predictive power, *P* between the inaccuracy provided by the PN and the actual error was evaluated using the MGE measures as follows:

(4) $$\begin{array}{c} P=\text{CORR}\left(\sum_{\text{path}}w_{i},\text{MG}{\text{E}}_{i}\right), \end{array}$$

where ∑_path_ *w*_*i*_ represents the sum of the weights for subject *i* according to a specific *path*, while MGE_*i*_ represents the actual error for the same subject *i*. Therefore the registration error predicted by the net was compared to the measures obtained on the data in order to assess its predicting power. The median values of *P* for the different datasets are 0.989, 0.985, and 0.998 for *R*_*c*_, *R*_*p*_, and SIM, respectively.

As it was reasonable to expect, the best correlation, that is, the best prediction, was obtained on simulated data. However, it is also convincing for real data.

The registration error was finally calculated in terms of intensity-based metrics for all datasets. In order to assess the predictive power of the MGE with respect to these other metrics, the correlation *C* between the inaccuracy measure provided using the MGE and each of the intensity-based metrics was calculated as follows:

(5) $$\begin{array}{c} C=\text{CORR}\left(M_{i}^{k},\text{MG}{\text{E}}_{i}\right), \end{array}$$

where *M*_*i*_^*k*^ represents the *k*th intensity-based metric (NMI, NRMS, and EO) calculated for the *i*th subject. Results revealed a higher correlation and a lower dispersion among the metrics for simulated data, while the opposite trend was observed for pathologic ones.

## 5. Discussion

In this paper the predicting power of the PN-based framework was proved, even when using for training only simulated data. Despite the simplified case that was considered, the proposed approach allowed to highlight some facts that are relevant at a system level as discussed above. In particular, it was possible to conclude that affine registration does not significantly improve the performance of rigid registration in the case of simulated and real adult data. Therefore rigid registration could then be safely chosen when computational efficiency is an issue. Following the same line, the straightforward application of the proposed method to more complex scenarios (such as multimodal registration and soft tissues) will enable the optimization of the pipeline by detecting and objectively assessing the main bottlenecks and exploiting this for guiding the pipeline shaping on the task at hand. Additional experiments relying on more complex cases are currently under investigation, and we foresee to further study the validation of longitudinal image registration.

Moreover, considering the optimization step, all the datasets were coherent in saying that this was a critical aspect in the registration pipeline. Regarding the choice of the interpolation scheme, while the difference in terms of error between the linear and the B-Spline method was not significant, NN interpolation appeared to negatively impact on the accuracy of the registration, as it was reasonable to expect. Simulated data proved to have a high predicting power with respect to real data in terms of absolute registration error. These results support the exploitation of simulated data to gain first insights on the behaviour of a registration system on real data. Finally, different types of metrics were compared to evaluate their respective relationships providing evidence of the coherent behaviour between the different types of metrics. In addition, the proposed framework is open and flexible and allows run-time monitoring of the accuracy level, as shown by its application to the children datasets. Further investigation is needed in order to explain the residual differences between the results obtained on simulated and real datasets as well as to unveil the impact of different pathologies. Moreover in future works we will further widen this research by accounting for deformable and intersubjects registration.

For what concerns the impact of the pathology on the registration outcome, the evaluation of images of schizophrenic and autistic patients did not highlight any statistically significant difference in terms of results. We aim at studying more invasive pathologies, in terms of impact on the brain morphology, for example, tumors, in order to evaluate how they impact the final registration outcome.

## 6. Conclusions

In this paper, a novel concept of validation and accuracy assessment in medical image processing was proposed based on PNs. Based on the analysis of the critical aspects in the affine registration workflow, it was possible to estimate the overall system (in)accuracy. It has been demonstrated that is possible to correlate the prediction with the quantitative measures which holds a great potential impact on the actual exploitability of the registration pipeline in real clinical settings. This could potentially be of great help in the clinical practice as it would enable to predict the inaccuracy of the process* a priori* or, viceversa, to tune *ad hoc* the different steps of the considered process.

## Figures and Tables

**Table 1:** Registration functional blocks.

* D *	MI		
* T *	Rigid	Affine	
Opt	RSGD	1 + 1 EO	
IF	NN	Linear	B-Splines

MI: mutual information; RSGD: regular steps gradient descent optimisation method; EO: evolutionary optimization algorithm; NN: nearest neighbours interpolation.

**Table 2:** Data labeling; *j* = *c*, *p* for control and pathologic subjects, respectively.

SIM	Simulated data
*R*	Real data
SIM^*τ*^	Simulated training data
SIM^*t*^	Simulated testing data
*R*_*j*_^*τ*^	Real training data, *j* = *c*, *p*
*R*_*j*_^*t*^	Real testing data, *j* = *c*, *p*

**Table 3:** Comparison between the weights obtained from *τ* and *t*, for simulated and real datasets.

		*R*_*c*_^*τ*^	*R*_*c*_^*t*^	*R*_*p*_^*τ*^	*R*_*p*_^*t*^	SIM^*τ*^	SIM^*t*^
* T *	Rigid	0.541	0.770	0.935	1.087	0.845	1.646
	Affine	0.435	0.749	1.084	1.069	1.040	1.740

Opt	RSGD	−0.596	0.210	0.132	0.086	−1.249	−0.179
	1 + 1 EO	1.559	1.303	1.882	2.081	3.124	3.564

IF	Linear	0.352	0.698	1.102	1.019	0.732	1.594
	NN	0.774	0.880	0.974	1.253	1.430	1.918
	B-Spline	0.329	0.690	0.940	1.015	0.672	1.577

**Table 4:** Comparison between the weights (expressed in mm) obtained from *τ* and *t*, for simulated and real datasets.

	*R*_*c*_^*τ*^	*R*_*c*_^*t*^	*R*_*p*_^*τ*^	*R*_*p*_^*t*^	SIM^*τ*^	SIM^*t*^
*R*_*c*_^*τ*^	1	0.999	0.954	0.994	0.995	0.989
*R*_*c*_^*t*^	0.999	1	0.966	0.998	0.998	0.994
*R*_*p*_^*τ*^	0.954	0.966	1	0.977	0.972	0.985
*R*_*p*_^*t*^	0.994	0.998	0.977	1	0.997	0.998
SIM^*τ*^	0.995	0.998	0.972	0.997	1	0.996
SIM^*t*^	0.989	0.994	0.985	0.998	0.996	1
